# Risk factor profiles for depression following childbirth or a chronic disease diagnosis: case–control study

**DOI:** 10.1192/bjo.2022.586

**Published:** 2022-10-07

**Authors:** Bradley S. Jermy, Saskia Hagenaars, Jonathan R. I. Coleman, Evangelos Vassos, Cathryn M. Lewis

**Affiliations:** Social, Genetic and Developmental Psychiatry Centre, Institute of Psychiatry, Psychology & Neuroscience, King's College London, UK; and NIHR Maudsley Biomedical Research Centre, South London and Maudsley NHS Trust, London, UK; Social, Genetic and Developmental Psychiatry Centre, Institute of Psychiatry, Psychology & Neuroscience, King's College London, UK; Social, Genetic and Developmental Psychiatry Centre, Institute of Psychiatry, Psychology & Neuroscience, King's College London, UK; NIHR Maudsley Biomedical Research Centre, South London and Maudsley NHS Trust, London, UK; and Department of Medical & Molecular Genetics, Faculty of Life Sciences and Medicine, King's College London, UK

**Keywords:** Depressive disorders, bipolar affective disorders, genetics, epidemiology, nosology

## Abstract

**Background:**

Progress towards understanding the aetiology of major depression is compromised by its clinical heterogeneity. The variety of contexts underlying the development of a major depressive episode may contribute to such heterogeneity.

**Aims:**

To compare risk factor profiles for three subgroups of major depression according to episode context.

**Method:**

Using self-report questionnaires and administrative records from the UK Biobank, we characterised three contextual subgroups of major depression: postpartum depression (3581 cases), depression following diagnosis of a chronic disease (409 cases) and a more typical (named heterogeneous) major depression phenotype excluding the two other contexts (34 699 cases). Controls with the same exposure were also defined. We tested each subgroup for association with the polygenic risk scores (PRS) for major depression and with other risk factors previously associated with major depression (bipolar disorder PRS, neuroticism, reported trauma in childhood and adulthood, socioeconomic status, family history of depression, education).

**Results:**

Major depression PRS was associated with all subgroups, but postpartum depression cases had higher PRS than heterogeneous major depression cases (OR = 1.06, 95% CI 1.02–1.10). Relative to heterogeneous depression, postpartum depression was more weakly associated with adulthood trauma and neuroticism. Depression following diagnosis of a chronic disease had weaker association with neuroticism and reported trauma in adulthood and childhood relative to heterogeneous depression.

**Conclusions:**

The observed differences in risk factor profiles according to the context of a major depressive episode help provide insight into the heterogeneity of depression. Future studies dissecting such heterogeneity could help reveal more refined aetiological insights.

Major depressive disorder (MDD) is a common psychiatric disorder with a lifetime prevalence between 6.5% and 21%.^1^ Heterogeneity in the clinical presentation of MDD includes severity and duration of each episode, differential symptomatology, number of episodes experienced and response to treatment.^2^ Clinical heterogeneity and a poor rate of treatment response^3,4^ argue for subgroups of depression which may benefit from specific treatments and have specific epidemiological and genetic risk factors.^5^

Historically, MDD was split into two broad classes, reactive and endogenous depression, which separate patients by a contextual explanation for their depressive episode. Following the publication of DSM-III, this separation was removed owing to lack of evidence for differences in outcome between the two diagnoses.^6,7^ Wakefield & Horwitz have argued that the current definition of MDD pathologises normal sadness by not adjusting the criteria when a state of low mood may be expected, for example following job loss or divorce. Cases in these contexts are less likely to reach the standard of a mental disorder according to a ‘harmful dysfunction’ theory, which requires a disorder to result from an impairment to an intrinsic biological or psychological mechanism.^8^ Indeed, there is evidence that clinicians already consider context when choosing treatment options.^9^

Two common contexts behind a major depressive episode are depression in response to a chronic disease and postpartum depression (PPD). The prevalence of MDD in general hospital in-patients is 12%^10^ and that of PPD in mothers with no prior history of mental illness is 17%;^11^ both rates are higher than the MDD point prevalence in the general population (4.7%).^12^

Many research studies use the term major depression as a broader definition than MDD, where cases may not be identified using structured clinical interviews and therefore may not attain clinical case status.^13^ The twin heritability of major depression is substantial, with estimates ranging between 20% and 50%.^14^ Genome-wide association studies (GWAS) indicate a highly polygenic architecture for major depression, where many common variants of small effect each confer risk.^13^ Polygenic risk scores (PRS), which capture an individual's genetic liability to major depression, show the genetic relationship between major depression and chronic diseases. For example, PRS for major depression were not associated with autoimmune disorders despite epidemiological associations.^15^ In a modestly sized study, PPD, but not major depression, was associated with PRS for bipolar disorder (BPD), suggesting a possible biological relationship between BPD and PPD.^16^

Being diagnosed with a chronic disease or having a new-born baby both require a sudden adaptation to a new environment. A chronic disease may require learning to live with an increased level of disability and possibly facing one's mortality; the postpartum period requires taking on responsibility for another human life, compounded with sleep deprivation. A period of sadness could be expected in both instances, and a milder version of PPD, ‘baby blues’, occurs in approximately 85% of mothers.^17^ Wakefield & Horwitz's harmful dysfunction theory leads to the hypothesis that these subgroups of MDD may have distinct biological and psychological mechanisms and might be less influenced by genetic risk factors.

In this study, we defined major depression by context of the depressive episode in the UK Biobank.^18^ We stratify major depression cases into three subgroups: depression diagnosed after diagnosis of a chronic disease, postpartum depression and a typical heterogeneous definition of major depression excluding the two other contexts. To dissect the differential risk factor profiles for these subgroups, we characterised their associations with known epidemiological risk factors and polygenic risk scores for major depression.

## Method

### Data

The authors assert that all procedures contributing to this work comply with the ethical standards of the relevant national and institutional committees on human experimentation and with the Helsinki Declaration of 1975, as revised in 2008. All procedures involving human participants/patients were approved by the North West Centre for Research Ethics Committee (11/NW/0382). This study was granted access to UK Biobank data after registration under approved application 18177. Written informed consent was available for all UK Biobank participants. The UK Biobank recruited 502 655 participants aged 40–69 in 2006–2010.^18^ To define major depression cases and subgroups, we combined self-report data (nurse interviews at baseline assessment and the Mental Health Questionnaire (MHQ) completed online by 157 366 participants in 2016–17)^19^ with administrative data from Hospital Episode Statistics (HES) and primary care records. Full details are given in the supplementary material (section Supplementary methods) available at https://doi.org/10.1192/bjo.2022.586.

### Heterogeneous depression

**Fig. 1:**
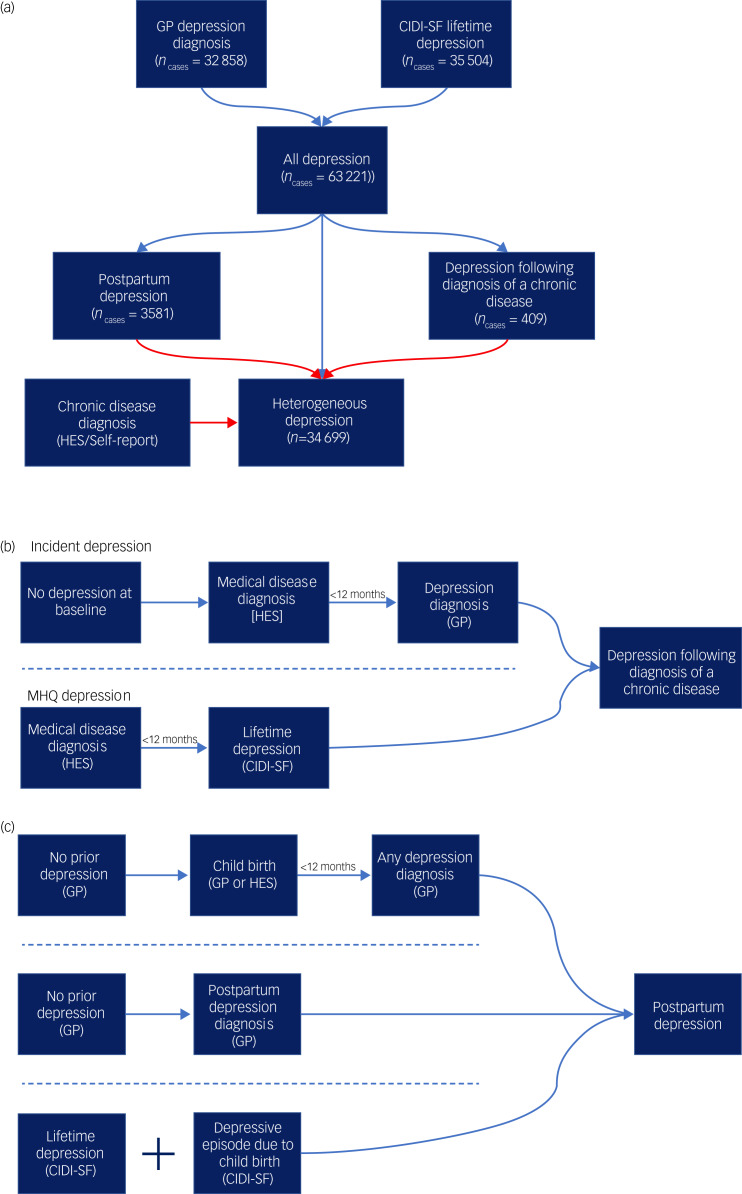
Defining the contextual definitions of major depression. (a) A flowchart for the three definitions of major depression used in the study. All diagnoses represent the union of two major depression definitions from self-report and primary care (general practitioner, GP) data. Cases are then allocated to one of three definitions: postpartum depression, depression following diagnosis of a chronic medical disease or heterogeneous depression. Arrows in red represent exclusion criteria. Cases of postpartum depression as well as any cases that report a chronic disease considered in this study are therefore removed from the definition of heterogeneous depression. (b) A summary schematic of the requirements a participant must meet to be designated case status for depression following diagnosis of a chronic disease. (c) A summary schematic of the requirements a participant must meet to be designated case status for postpartum depression. In parts (b) and (c), arrows represent the flow of time and dashed lines separate qualifying criteria. For both depression following diagnosis of a chronic disease and postpartum depression only one set of criteria separated by the dashed lines is required to attain case status. HES, Hospital Episode Statistics; CIDI-SF, Composite International Diagnostic Interview – Short Form (taken from the Mental Health Questionnaire).

Cases of major depression were defined using the Composite International Diagnostic Interview – Short Form (CIDI-SF) within the MHQ and/or a primary care code specific to depression (see supplementary Table 1 for a list of primary care codes). Cases were allocated to the contextual major depression definitions of postpartum depression and depression following diagnosis of a chronic disease, then heterogeneous depression was defined as major depression cases not allocated to either context (Fig. 1(a)).

### Depression following diagnosis of a chronic disease

This phenotype is defined as participants experiencing their first major depressive episode within a year of receiving a diagnosis of a chronic disease. We selected a subset of diseases associated with increased risk of major depression (stroke,^20^ diabetes,^21^ autoimmune disorders,^22^ cancers (excluding non-melanoma skin cancers),^23^ motor neuron disease,^24^ myocardial infarction^25^) and extracted the date of first diagnosis (see supplementary Table 2 for a list of ICD codes and definitions used for each chronic disease).

The final phenotype represents the union of cases across two definitions – incident depression and MHQ depression. Incident depression cases were participants who did not report major depression at baseline and subsequently had their first major depression diagnosis, according to administrative data (Supplementary methods, supplementary Tables 1 and 3). Cases were retained if depression onset was within 12 months of a chronic disease diagnosis (Fig. 1(b)).

Cases of MHQ depression were defined using the CIDI-SF^26^ following DSM criteria for an individual's worst episode of depression. The reported age of their first major depressive episode was matched against the age at onset for each of the chronic diseases. Cases were retained if the two ages were equal or the first major depressive episode was up to 1 year later.

### Postpartum depression

Cases of PPD were identified from three UK Biobank data sources (Fig. 1(c)):

- major depression cases from the MHQ CIDI-SF where it was confirmed that the worst depressive episode was ‘possibly related to childbirth’ (field 20445)
- a primary care record of PPD (read codes: 62T1, Eu530) with no earlier records of depression
- a primary care record of the earliest date of major depression diagnosis within 1 year of giving birth.

### Definition of controls and exclusion criteria

For each depression subgroup, we defined controls having the same exposure as the cases: controls for PPD must have given birth, controls for depression following a chronic disease must have been diagnosed with one of the chronic diseases, and controls for the heterogeneous definition of depression must not have reported any of the chronic diseases. Control groups were screened for multiple definitions of major depression. Cases and controls were excluded if they reported a diagnosis of, or medication for, psychosis, bipolar disorder or substance misuse.

### Risk factors

Six epidemiological risk factors previously associated with major depression were selected:^27^ education, socioeconomic status (SES), neuroticism, family history of severe depression,^28^ reported childhood trauma^29^ and reported adulthood trauma.^30^ Education is a four-level categorical variable with qualifications attained at (a) college or university (the reference group), (b) age 18 (A-levels or equivalent), (c) age 16 (General Certificate of Secondary Education (GCSE) or equivalent) and (d) no reported qualifications. SES was measured using the regional Townsend Deprivation Index (TDI) and neuroticism was the sum score of the Eysenck Personality Inventory Neuroticism scale (EPIN-R).^31^ Sum scores are justified by the high internal consistency and replicated factor structure of the EPIN-R,^32^ as well as ensuring a phenotype consistent with previous literature.^33,34^ For trauma variables, items from a 16-item trauma questionnaire were assigned to childhood or adulthood. Summary trauma measures were defined as the first principal components^35^ of the tetrachoric correlation matrix,^36^ separately for reported childhood and adulthood trauma. These components explained 55.0% and 27.5% of the variance in reported childhood and adulthood trauma respectively (supplementary Fig. 1 gives a breakdown of each item's contribution to the principal component).

For PRS, a previously defined pipeline for genetic quality control was applied to retain unrelated participants of European ancestries.^18,37^ PRSice version 2 (for Linux)^38,39^ was used to generate PRS for major depression and BPD using summary statistics from Wray et al^13^ (*n*_cases_ = 116 404, *n*_controls_ = 314 990; UK Biobank samples removed) and Stahl et al^40^ (*n*_cases_ = 20 352, *n*_controls_ = 31 358) respectively. *P*-value thresholds (*P*_t_) previously shown to optimise prediction were selected (major depression PRS: *P*_t_ < 0.05;^13^ BPD PRS: *P*_t_ < 0.01^40^) and a sensitivity analysis was performed including all single nucleotide polymorphisms (SNPs) (*P*_t_ ≤ 1).

### Statistical analysis

Complete case logistic regression was used to test for association between case–control status under each major depression definition and each risk factor. For analysis of epidemiological risk factors, the covariates of assessment centre, education, TDI and year of birth were included. Regression models used to assess education and TDI are therefore nested within the models for other epidemiological risk factors and have a slightly different interpretation – namely, the effects reported for epidemiological risk factors are independent of education and TDI. To account for population stratification and technical artefacts, six genetic principal components, genotyping batch and assessment centre were included as covariates for PRS regressions.^41^ Given that PRS, neuroticism and TDI scores have no inherent meaning, the variables were standardised before analysis so that each variable can be interpreted as the effect per standard deviation of the population.

To determine whether the effect size for a risk factor differed between major depression subgroups, odds ratios (ORs) for PPD and for depression following a chronic disease were compared with heterogeneous depression using the Wald test.

A case–case analysis was performed to understand heterogeneity among major depression cases, comparing the heterogeneous definition of depression with PPD and with depression following a chronic disease for each risk factor.

Sensitivity analyses were performed by source of major depression diagnosis from primary care or self-report, and for PPD using only female participants for the heterogeneous definition.

The Benjamini–Hochberg false discovery rate (FDR) method was used to correct for multiple testing (*q* < 0.05).^42^ Correction was performed separately for the set of case–control, case–case and female-only regressions and Wald tests, as each answer a different research question with distinct interpretations.

## Results

### Phenotypes

Analysis of UK Biobank identified 3581 cases for PPD, 409 cases for depression following a chronic disease, and 34 699 cases for heterogeneous depression. Sample characteristics of each group are shown in Table 1, with risk factor missingness and self-reported ethnicity in supplementary Tables 4 and 5.

**Table 1:** Distribution of values in epidemiological and genetic risk factors across three definitions of major depression^a^

Variable	Heterogeneous depression		Postpartum depression		Chronic disease depression	
	Control	Case	Control	Case	Control	Case
SES, mean (s.d.)	−1.61 (2.93)	−1.27 (3.05)	−1.68 (2.88)	−1.70 (2.81)	−1.33 (3.08)	−1.35 (2.97)
Neuroticism mean (s.d.)	3.00 (2.75)	5.69 (3.37)	3.46 (2.77)	5.92 (3.26)	3.09 (2.76)	4.42 (3.02)
Adult trauma (PC1), mean (s.d.)	0.31 (0.38)	0.57 (0.55)	0.36 (0.45)	0.70 (0.64)	0.35 (0.39)	0.51 (0.47)
Child trauma (PC1), mean (s.d.) (PC1)	0.21 (0.36)	0.42 (0.53)	0.21 (0.39)	0.50 (0.58)	0.23 (0.39)	0.33 (0.51)
Family history of severe depression	Endorsed by 5.96% (No: 69 264; Yes: 4387)	Endorsed by 14.97% (No: 29 032; Yes: 5113)	Endorsed by 6.85% (No: 106 412; Yes: 7830)	Endorsed by 18.05% (No: 2888; Yes: 636)	Endorsed by 5.92% (No: 61 553; Yes: 3875)	Endorsed by 12.81% (No: 347; Yes: 51)
Education	Degree: 28 624 (39%), A-levels: 24 083 (33%), GCSE: 12 982 (18%), None: 8327 (11%)	Degree: 13 680 (40%), A-levels: 11 480 (33%), GCSE: 5455 (16%), None: 3661 (11%)	Degree: 33 953 (30%), A-levels: 35 960 (31%), GCSE: 23 419 (20%), None: 21 372 (19%)	Degree: 1400 (40%), A-levels: 1304 (37%), GCSE: 586 (17%), None: 241 (7%)	Degree: 18 091 (27%), A-levels: 21 398 (32%), GCSE: 10 736 (16%), None: 15 793 (24%)	Degree: 134 (33%), A-levels: 133 (33%), GCSE: 63 (16%), None: 71 (18%)

SES, socioeconomic status; PC1, principal component 1.

a. Values reported represent the raw scores (variables have not been standardised). As education and SES were used as a covariate in each regression, distributions of all other variables are following removal of samples that had a missing value for either education or SES.

### Polygenic risk scores

**Fig. 2:**
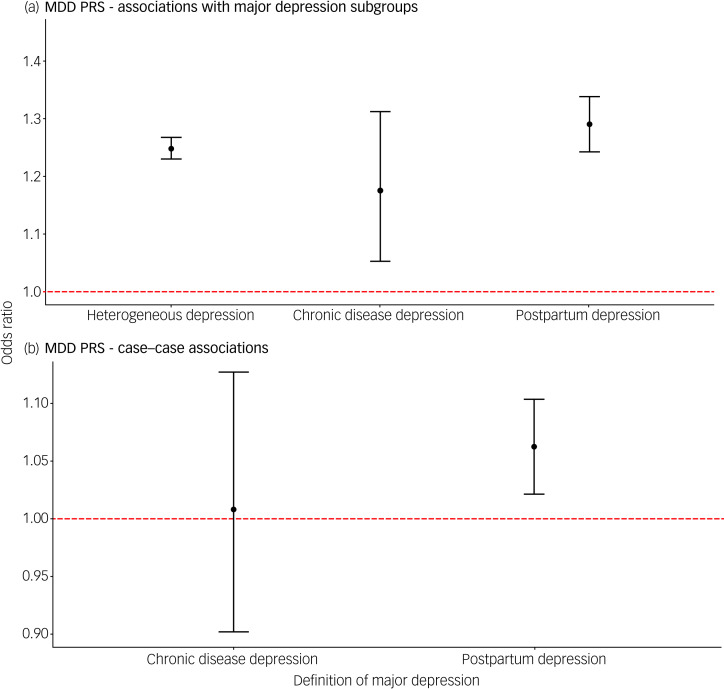
(a) Associations of polygenic risk score for major depression with three contextual subgroups of major depression. (b) Associations of polygenic risk score for major depression comparing cases of postpartum depression and depression following a chronic disease with a heterogeneous definition of depression. In each test, heterogeneous depression is the reference group. Error bars in both panels represent 95% confidence intervals. The dashed red line represents the point at which the risk factor shows no association with the contextual definition of major depression (odds ratio = 1).

All PRS were standardised within each group, so odds ratios represent the change in odds of being a case per one standard deviation increase in PRS. The PRS for major depression was associated with each depression phenotype compared with controls (heterogeneous depression: OR = 1.25, 95% confidence interval (CI) 1.23–1.27; depression following a chronic disease: OR = 1.18, 95% CI 1.05–1.31; PPD: OR = 1.29, 95% CI 1.24–1.34; Fig 2(a)). Relative to heterogeneous depression, PPD and depression following a chronic disease had comparable effect sizes.

The PRS for BPD was also associated with each depression definition, with comparable estimates across groups (heterogeneous depression: OR = 1.09, 95% confidence interval (CI) 1.07–1.11; depression following a chronic disease: OR = 1.16, 95% CI 1.04–1.30; PPD: OR = 1.09, 95% CI 1.05–1.14; supplementary Fig. 2). Similar effect size estimates were obtained from the sensitivity analysis using PRS with all SNPs (*P*_t_ ≤ 1) (supplementary Fig. 3), but there was evidence of a stronger association of the BPD PRS when using electronic health records to define PPD (supplementary Fig. 4, supplementary Table 6).

We performed case comparisons to determine whether the context of a depressive episode indexed heterogeneity within cases for the PRS for major depression and BPD. The case-only analysis removes variation between our control groups and therefore has a different interpretation from the case–control analyses above. The PRS for major depression was associated with PPD compared with heterogeneous depression (OR = 1.06, 95% CI 1.02–1.10; Fig. 2(b)). Associations with PPD remained significant after restricting heterogeneous depression to females only (supplementary Table 7). No differences were found between groups for BPD PRS (supplementary Table 8, supplementary Fig. 5).

### Risk factors

**Fig. 3:**
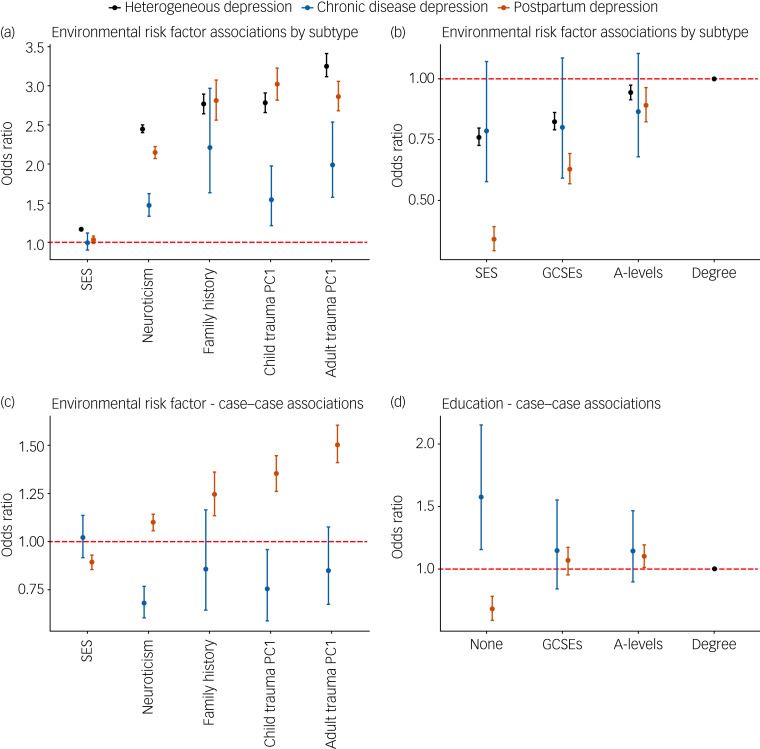
(a) Association of three contextually based subgroups of major depression with epidemiological risk factors. (b) Association of three contextually based subgroups of major depression with education; having a college or university degree is the reference category. (c) Case–case comparisons for epidemiological risk factors. (d) Case–case comparison for educational attainment. In each case–case comparison, cases of heterogeneous depression are the reference group, with all effects of educational attainment being relative to attaining a college or university degree. For all graphs error bars represent 95% confidence intervals. The dashed red line represents the point at which the risk factor shows no association with the contextual definition of major depression (odds ratio = 1). PC1, principal component 1. Supplementary Fig. 6 displays the same associations using log odds to allow for a linear comparison between risk factors and their differences between contextual subgroups.

In general, the risk factors were significantly associated with each major depression definition. Depression following a chronic disease was not associated with SES, as measured by TDI, or education. PPD was not associated with SES (Fig. 3(a–b); supplementary Table 9).

### Depression following a chronic disease

Relative to heterogeneous depression, neuroticism, SES and reported childhood and adulthood trauma had weaker associations with depression following a chronic disease (Fig. 3(a); supplementary Table 9). Comparing cases of depression following a chronic disease with heterogeneous depression revealed that the former case group had lower neuroticism scores, were less likely to report childhood trauma and were more likely to report not having any qualifications compared with a degree (Fig. 3(c) and (d); supplementary Table 8).

### Postpartum depression

The odds ratios for SES, neuroticism and reported adulthood trauma were lower for PPD relative to heterogeneous depression. Higher education was associated with greater risk of PPD, with a larger dose response shown relative to heterogeneous depression (Fig. 3(a) and (b); supplementary Table 9). Sensitivity analyses show that this association with education is largely driven by the MHQ definition of PPD (supplementary Fig. 4, supplementary Table 6). In PPD, the differences in odds ratios for reported adulthood trauma did not survive multiple testing in analysis of the female subset of heterogeneous depression (supplementary Table 10).

The case–case comparison, which removes any heterogeneity in control definitions, showed increased neuroticism scores, family history of severe depression, and reporting of childhood and adulthood trauma among PPD cases compared with cases of heterogeneous depression (Fig. 3(c); supplementary Table 8). PPD cases had higher SES, were more likely to have attained A-levels and less likely to report no qualifications compared with a degree (Fig. 3(d); supplementary Table 8). The association with A-levels in PPD was attenuated when the analysis was repeated using female-only heterogeneous depression. Although most odds ratios remained consistent in this reanalysis, reports of adulthood trauma reduced from 1.51 to 1.26 (supplementary Table 7).

## Discussion

We sought to understand the role of context in depression by showing differential associations with genetic and epidemiological risk factors in three major depression subgroups – depression following diagnosis of a chronic disease, postpartum depression and a non-contextual definition named heterogeneous major depression.

Risk factors of major depression PRS, BPD PRS, neuroticism, family history of severe depression and reported trauma in both childhood and adulthood were associated with major depression in each subgroup. Other epidemiological risk factors showed a more complex pattern: SES was associated with both PPD and heterogeneous major depression, whereas education was associated with heterogeneous major depression for all levels and PPD for most levels (see Supplementary discussion for a deeper explanation of our findings on education). The association across contextual subgroups suggests that these risk factors remain important to the aetiology of major depression, independent of the context of diagnosis. If the PRS for major depression becomes sufficiently predictive to be implemented as a clinical decision tool for depression, the comparable associations across contextual subgroups suggest there will be a minimal discrepancy in utility. However, this requires our findings to be replicated in future, more powerful, studies.

Depression following a chronic disease had weaker associations with SES, neuroticism, and reported adulthood and childhood trauma compared with heterogeneous depression. Low SES is associated with poorer physical and mental health.^43^ Controls with a chronic disease had lower SES than controls for heterogeneous depression (supplementary Table 11), reducing the association between SES and depression following a diagnosis of a chronic disease.

Trauma and neuroticism may play a weaker role in depression when faced with the immediate stress of being diagnosed with and living with a chronic disease (Supplementary discussion). This assumes that the chronic disease is the primary reason for the depressive episode and would imply disease-related risk factors for this depression subgroup. Indeed, it has been shown that a person's level of disability after a stroke,^44^ physical capabilities of people with type 2 diabetes^45^ and disease course in multiple sclerosis^46^ are all associated with major depression.

Case–case comparisons between PPD and heterogeneous depression showed opposite directions of association relative to case–control comparisons for neuroticism and reported adulthood trauma. This effect likely arises from heterogeneity within the control groups. For adulthood trauma, it may be driven by the female-only control group. Women are more likely to report interpersonal traumatic experiences, including sexual assault,^47^ which is an important component of adulthood trauma. Indeed, PPD controls had higher levels of reported adulthood trauma than heterogeneous depression controls (supplementary Table 11), and the female-only analysis of heterogeneous depression attenuated the association (supplementary Table 10). The same was shown for neuroticism, with controls for PPD having higher neuroticism scores. Heterogeneity in control groups further highlights the importance of appropriate comparisons. Without subgrouping, meaningful differences can be masked, which may help to inform stratified treatments.

PPD also shows a weaker association with SES. This may reflect a more equitable distribution of health services during the postpartum period. Standardised implementation of early detection and management of depressive symptoms in new mothers may reduce the disparity in prevalence associated with deprivation. Although there is some evidence to support that this is the case for deprivation, inequalities remain, particularly in ethnic minorities.^48^

In PPD, we found no evidence for a stronger association with BPD PRS relative to major depression PRS, and did not replicate the findings from Byrne et al.^16^ Subsequent replication studies have consistently shown an association between major depression PRS and PPD,^49–52^ suggesting that the lack of association in Byrne et al^16^ may have been due to the use of a GWAS with insufficient power. Regarding BPD PRS, some studies have found associations with PPD^51,52^ but others have not.^49,50^ Interestingly, the opposite was found in two studies, with major depression PRS being more strongly associated than BPD PRS.^50,52^ Further, this study and Byrne et al^16^ differ in methodology, sample characteristics and phenotype definitions, all of which could influence replicability. Indeed, we find evidence that the data used to define the PPD phenotype influences the strength of association, showing a stronger association with administrative records relative to self-report data (supplementary Fig. 4, supplementary Table 6). Understanding the heterogeneity in association across data types was beyond the scope of this paper but it warrants a systematic analysis to improve future phenotyping efforts.

Taken together, these results highlight the importance of considering the likelihood of a depressive episode given the context the participant currently faces. Comparisons across subgroups of depression could provide translational benefit through pointing to risk factors of major depression that are directly related to the response to the specified context. Given that controls also differ between subgroups, heterogeneity within cases was explored in a case–case analysis which directly tests whether aggregating major depression cases can mask associations with a risk factor. As with the subgroup analysis, reported childhood trauma and neuroticism scores were decreased in cases of depression following a chronic disease, indicating that the prior results are driven partly by heterogeneity within cases. A control–control comparison showing heterogeneity within controls also contributes to our results (supplementary Table 11). We additionally show an effect of education whereby people with depression following a chronic disease were more likely to report having no recognised UK-based qualifications. Given the association of physical health with SES and that a key separation between the two groups is the requirement for a chronic disease, it is possible that this finding is an SES effect.

Previous studies consistently find childhood trauma to predict PPD, as summarised by a systematic review by Segura et al.^53^ PPD has been identified as a mediator in the relationship between reported trauma and mother–infant relationships postpartum, highlighting the important role treatment can have on both mother and child.^54^ The literature is less clear, however, regarding our finding of an increased association in PPD relative to heterogeneous depression. A possible explanation is that becoming a parent leads to greater reflection on both the positive and negative aspects of one's childhood. This could increase the likelihood of parents finding links between their depressive episode and trauma suffered during childhood relative to non-parents, thus representing mood-congruent recall biases.^55^ It is important to stress that trauma was assessed retrospectively, which may differ from prospectively measured trauma.^56^

Major depression PRS was elevated in PPD cases relative to heterogeneous depression cases, a finding corroborated by an increased reporting of family history of severe depression. This finding replicates a recent study by Kiewa et al (2021) which used the Australian Genetics of Depression Study to show that cases for PPD have elevated genetic risk compared with cases for major depression excluding PPD.^52^ Biological theories often attribute PPD to hormonal changes that occur during the perinatal period. A recent study shows that genetically predicted levels of C-reactive protein (CRP) are associated with PPD,^51^ supporting a previous finding that CRP levels before or immediately during the postpartum period predict PPD.^57^ A systematic review, however, shows that evidence for such biological theories is inconsistent.^58^ Although some studies suggest a role for corticotropin-releasing hormone trajectories during pregnancy,^59,60^ most studies have small samples, are inconsistent in their definition of PPD and do not account for environmental confounders. Our finding suggests that biological variation is an important component and that focusing on PPD could improve power in future genetic research for this phenotype. Determining whether PPD has distinct biological pathways will require targeted GWAS such as the ‘Mom Genes’ study.^61^

### Limitations

The findings presented should be considered in light of the following limitations. First, although this study dissects heterogeneity of depression via context, the subgroups themselves remain heterogeneous. For each subgroup, we were unable to determine the severity of the depressive episode, which may have contributed to our results. Similarly, the decision to implement a 12-month cut-off for PPD and depression following a chronic disease is arbitrary.

Second, for depression following a chronic disease, we aggregated across multiple diseases because of sample size constraints. Our hypothesis was generated through the lens of individual differences in psychological resilience after receiving a diagnosis; resilience may differ by disease and by severity, which we were unable to explore. Other explanations suggest that depressive symptoms may arise through shared biology or as side-effects of disease treatment.^62^

Third, our phenotypes are derived from self-report questionnaires and electronic health records, which each have limitations. Retrospective self-report definitions are subject to recall bias, increasing misclassification of both cases and controls. Electronic health records are prospective but may not represent a complete record of a participant's medical history. Indeed, HES data relating to psychiatry were not present for Scottish participants, meaning that potential cases of major depression are missed. Similarly, primary care data are currently available for only approximately 230 000 individuals. Some participants may have experienced a depressive episode without seeking help from a general practitioner. We quantified the effect of data source, showing that effect sizes for most risk factors are comparable between phenotypes of PPD using self-report and electronic health records. Cohort effects from changing prevalence of depression across time may also be present. Portability of PRS across ancestries is poor,^63^ and our PRS analysis was restricted to those of European ancestry.

Fourth, neuroticism, childhood trauma and adulthood trauma had a high degree of missingness in the sample (supplementary Table 4), due either to endorsing ‘prefer not to answer’ or ‘don't know’ for individual items, or to not participating in the follow-up Mental Health Questionnaire. It is likely such responses are missing not at random,^64^ which could introduce selection bias into our associations as we have performed a complete-case analysis.^65^ Given the nature of the missingness, it is plausible that it is partly due to the question, which limits imputation accuracy.^64^ While acknowledging the limitation of a complete-case analysis, we believe that the alternative of imputation holds a similar if not greater risk of bias. Related to this, it would have been of interest to perform a multiple regression with all risk factors included, but the varying degrees of missingness per variable and, therefore, the potential to introduce bias, complicates interpretation relative to assessing epidemiological risk factors independently.

Fifth, our summary measures for trauma reported in childhood and adulthood explained 55% and 27.5% of the total variation respectively. Although we believe that these components capture sufficient information to gain a general understanding of the interplay between trauma and our depression phenotypes, it does leave variation unexplored. A deeper analysis, at the item level for the trauma questionnaire, may reveal some interesting findings for future studies.

Finally, index event bias is a form of collider-stratification bias that occurs when a sample is stratified according to an event that is strongly associated with the risk factor and outcome. Such a bias may therefore manifest in our study design; however, as reasoned in the supplementary discussion (supplementary Table 12), we believe that our conclusions are unlikely to have been materially affected.

## Data Availability

The data that support the findings of this study are available to all researchers with an appropriate UK Biobank application. The necessary scripts to reproduce the analysis can be found at https://github.com/Bjermy/contextualdepression.

## Supplementary material

For supplementary material accompanying this paper visit http://doi.org/10.1192/bjo.2022.586. click here to view supplementary material
